# Joint Center Estimation Using Single-Frame Optimization: Part 1: Numerical Simulation

**DOI:** 10.3390/s18041089

**Published:** 2018-04-04

**Authors:** Eric Frick, Salam Rahmatalla

**Affiliations:** 1 Center for Computer-Aided Design, College of Engineering, The University of Iowa, Iowa City, IA 52242, USA; eric-frick@uiowa.edu; 2 Department of Civil and Environmental Engineering, College of Engineering, The University of Iowa, Iowa City, IA 52242, USA

**Keywords:** motion capture, inertial sensors, skin motion, optical markers, soft tissue artifact

## Abstract

The biomechanical models used to refine and stabilize motion capture processes are almost invariably driven by joint center estimates, and any errors in joint center calculation carry over and can be compounded when calculating joint kinematics. Unfortunately, accurate determination of joint centers is a complex task, primarily due to measurements being contaminated by soft-tissue artifact (STA). This paper proposes a novel approach to joint center estimation implemented via sequential application of single-frame optimization (SFO). First, the method minimizes the variance of individual time frames’ joint center estimations via the developed variance minimization method to obtain accurate overall initial conditions. These initial conditions are used to stabilize an optimization-based linearization of human motion that determines a time-varying joint center estimation. In this manner, the complex and nonlinear behavior of human motion contaminated by STA can be captured as a continuous series of unique rigid-body realizations without requiring a complex analytical model to describe the behavior of STA. This article intends to offer proof of concept, and the presented method must be further developed before it can be reasonably applied to human motion. Numerical simulations were introduced to verify and substantiate the efficacy of the proposed methodology. When directly compared with a state-of-the-art inertial method, SFO reduced the error due to soft-tissue artifact in all cases by more than 45%. Instead of producing a single vector value to describe the joint center location during a motion capture trial as existing methods often do, the proposed method produced time-varying solutions that were highly correlated (*r* > 0.82) with the true, time-varying joint center solution.

## 1. Introduction

In the 140-plus years since its inception, motion capture has grown from a novelty used to win bets into a ubiquitous tool for use in fields such as animation, healthcare, industry, sports, and the military [1,2,3,4,5]. Optical motion capture is currently considered the field’s gold standard, and it works by using cameras to visually deduce the position and posture of a subject who is covered in markers [6,7]. The paramount importance of accurate calculation of joint axes and joint centers in determining accurate joint kinematics has spawned much research into methods of using optical markers to determine them [8,9,10,11,12].

Determination of joint center locations can be accomplished via predictive methods, such as the Harrington Equations, where regression equations are applied to a subject’s anthropometric measurements [13]. Marker-based methods for direct computation of joint centers can be categorized as sphere-fitting or transformation approaches, or collectively as functional methods. Sphere-fitting approaches use optimization to determine a radius (and related sphere) that best fits the measured marker trajectories [8]. Transformation approaches consider the distance between markers on each joint segment to be fixed, allowing local coordinate systems to be defined. The measurements can then be mapped to a common reference frame, allowing an optimal joint center to be approximated [8]. For joint axis determination, the same general approaches as for joint center determination can be applied, but with cylinder-fitting instead of sphere-fitting, as the desired solution is a line (axis of rotation) rather than a point [9]. Although this area is generally dominated by marker-based approaches, recent publications involving inertial motion capture offer competing methods for joint axis determination. By applying optimization to expressions of joint kinematics, the joint axes for two-degree-of-freedom joints (such as the elbow) can be identified [14] and leveraged to mitigate the effects of magnetic interference in the inertial motion capture process [15].

These functional methods perform very well on mechanical apparatuses, but their accuracy decreases when applied to human subjects due to soft-tissue artifact (STA). STA allows skin-mounted markers to move relative to the underlying bone in a manner that is difficult to fully mitigate via modeling [16,17,18,19,20]. STA can be defined as the combination of four transformations: translation, rotation, scaling, and shearing, though more complex deformations are also possible [21,22,23]. STA can introduce significant errors in the joint center location calculated from markers when compared against gold-standard validation methods (e.g., X-ray and bone-pin markers). In a current article [16] on estimation of joint angle and range of hip motion using skin markers and dual fluoroscopy, it was found that the mean STA ranged from 0.3 to 5.4 cm. When calculating the hip-joint angle, STA caused errors (relative to the dual fluoroscopy validation method) in flexion/extension, abduction/adduction, and internal/external rotation of 1.9°, 0.6°, and 5.8°, respectively. Range of motion was also impacted, as the marker-based range of motion for the internal-external rotation axis was 21.8° smaller than that of the validation method. 

STA has been thoroughly investigated in recent literature [16,18,19,20,21,22,24,25]. The most recent research, by way of modal analysis or principal component analysis, shows that, of STA’s many components, the translation and rotation are likely the main determinants of kinematic accuracy [19,21,22]. Together, these two transformations constitute the rigid component of STA-induced motion. However, more recent research investigated STA with a greatly increased number of optical markers (40 versus 4–6) and found the observed STA to be more complex than indicated by previous research, sometimes requiring the use of stretching and homotheties to fully describe the behavior [23]. This suggests that modeling STA via only its rigid components may lead to incomplete and/or insufficient mitigation of STA’s effect on the accuracy of joint kinematics. In sum, the current literature suggests that motion capture accuracy could be improved by a practical and non-invasive method that more completely mitigates the effects of STA. 

In the last decade, inertial motion capture has emerged as another method of tracking human movement [26,27]. Inertial motion capture centers on magnetic, angular rate, and gravitational sensor arrays, often referred to as inertial measurement units (IMUs). The literature showed several methods that used IMUs in the analysis of human motion [28,29,30]. However, a recent survey on motion tracking using IMUs suggests that optimization-based approaches are more promising than other methods and offer considerable room for improvement [27]. Said optimization can be achieved via reformulating the equations of motion for translational acceleration of rotating bodies, allowing the position vector locating the IMU relative to the joint center to be calculated directly [28,31,32]. In the work of Seel et al., the relative alignment of the sensor and joint axis is optimized in a similar manner [31]. The subject first performs a specialized calibration motion to manifest the kinematic constraints associated with the optimization, allowing subject-specific joint centers and joints axes to be determined. Joint angles are calculated by a Kalman Filter–based integration process, producing results for knee flexion/extension accurate to within 3° of root mean square error relative to the marker approach used for validation [28].

This paper proposes a novel approach to joint center estimation applied via sequential application of single-frame optimization (SFO). The method attempts to mitigate the effects of STA by first minimizing the variance of individual time frames’ joint center solutions to obtain an accurate overall joint center estimate. This is accomplished via the developed variance minimization method. The resulting joint center estimate is then used as initial conditions to stabilize an optimization-based linearization of human motion that determines a time-varying joint center estimation. The proposed method, henceforth referred to as SFO, requires accelerometric and gyroscopic measurements only; magnetic data is not utilized. The method was tested in this work via a simulated mechanical system (rotating planar pendulum) with an inertial sensor attached to it. STA was simulated by allowing the inertial sensor to translate and rotate relative to the pendulum.

## 2. Methods 

### 2.1. Equations of Motion

IMUs can measure only derivatives, be they positional or rotational. Therefore, in order to relate the inertial measurements—translational acceleration and rotational velocity—to the joint center vector (in the local coordinate system of the inertial sensor), the following equation of motion can be used

(1) $$a=a_{JC}+\alpha \;\times \;r+\omega \;\times \;\left(\omega \;\times \;r\right)$$

Here a is the overall translational acceleration, $a_{JC}$ is the translational acceleration of the joint center, ω is the rotational velocity, α is the rotational acceleration, and r is the position vector describing the location of the IMU relative to the joint center. All these values are expressed within the local coordinate system of the IMU. The nature of this equation is such that it can be applied to joints modeled as having one, two, and/or three degrees of freedom. Notice that Equation (1) is valid only if ω and a are measured by a sensor rigidly connected to a link, and only if the link length (meaning r) has null values for its first and second time derivatives. In the context of human motion capture, STA ensures that neither of these conditions is satisfied. The inertial measurements will be contaminated by the translational acceleration, rotational velocity, and rotational acceleration caused by STA, and the distance from the IMU to the joint center will inevitably vary so that it has non-zero first and second derivatives, also due to STA. 

### 2.2. Spanned Single-Frame Optimization

SFO uses Equation (1) as the basis of its cost function, with the understanding that the model embedded in its cost function can only produce solutions pertaining to rigid-body motion. Said solutions will henceforth be referred to as rigid-body realizations. The vector relating an IMU to its corresponding joint center (represented by r) will necessarily vary with time in human movement due to the impact of STA. To track this motion, SFO calculates a rigid-body realization (meaning a value for r) for each individual frame of data. Defining the joint center vector as the time series of these rigid-body realizations allows the non-rigid nature of the STA-contaminated joint center vector to be accurately captured, effectively linearizing the joint center vector and its corresponding STA. SFO will therefore be presented as operating under two assumptions. First, the value of $a_{JC}$ is negligible relative to the other terms, and second, any acceleration terms generated by STA are negligible relative to those generated by the STA-free motion, henceforth referred to as real motion. With this in mind, Equation (1) can be rearranged into a cost function in terms of r that quantifies the optimality of a joint center vector estimate

(2) 0=||a−(α×r+ω×(ω×r))−g||

Here the double bars on either side denote the Euclidean norm, and g denotes the scalar value of gravitational acceleration. This scalar is subtracted because gravitational acceleration will impact the accelerometer measurements. Note that the $a_{JC}$ term has been removed in this equation as it is assumed that the origin of the position vector is undergoing no translational acceleration. Equation (2) can be modified to allow for an accelerating origin, but, for the purposes of this article, such modification has little heuristic value. The modified equation will instead be explored in a future paper.

SFO uses Equation (2) as a cost function and applies an optimization algorithm to each frame of data individually. This results in a time-varying realization for r over the course of the considered motion. The optimization method chosen for this work is the unconstrained Levenberg–Marquardt method with a supplied analytical Jacobian, which is part of the MATLAB Optimization Toolbox [33]. The Levenberg–Marquardt method was chosen for its robust solutions and intuitive methodology. The default properties built into the MATLAB function “fsolve” were used with one modification—the Levenberg–Marquardt method was specified as the optimization algorithm. The most relevant of these default values are the function tolerance and maximum allowed iterations, which were 1 × 10^−6^ and 400, respectively. The analytical Jacobian was included to expedite the optimization, but it had little effect on the final result.

**Figure 1:**
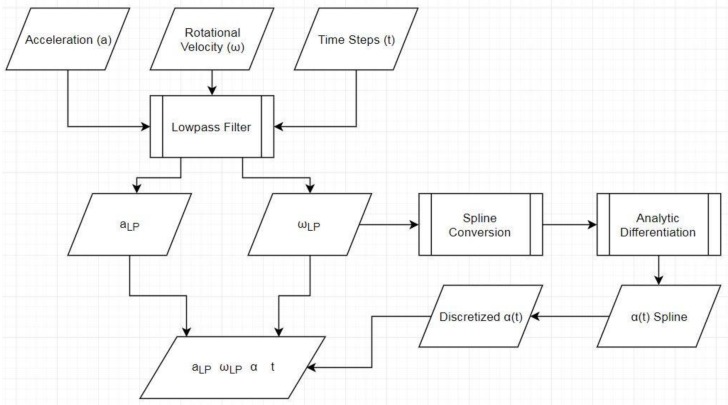
Flowchart detailing how the data is pre-processed before being fed into SFO. The subscript “LP” denotes that the variable has been lowpass filtered.

**Figure 2:**
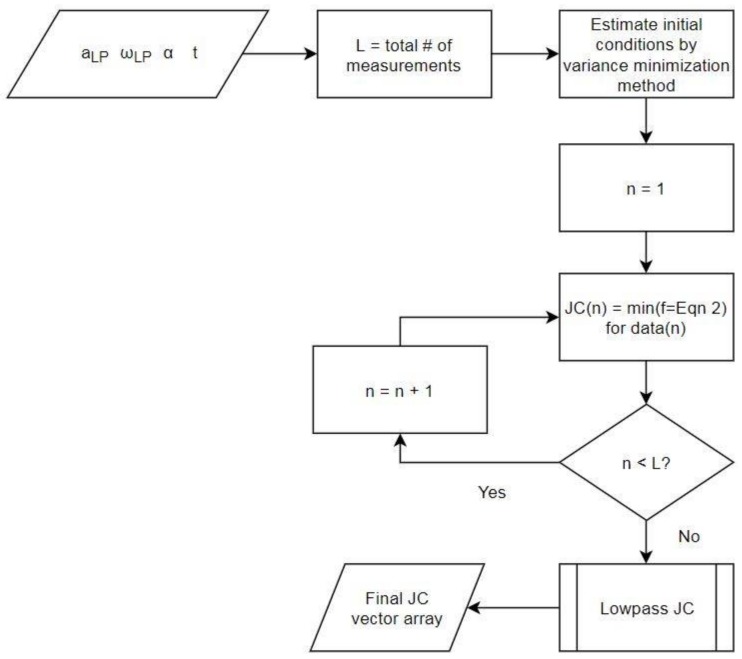
Flowchart representation of the SFO method. The subscript “LP” denotes that the variable has been lowpass filtered, “t” denotes the time vector, “JC” denotes joint center, and “Eqn” denotes Equation.

The first step in SFO is preparing the data (Figure 1), which involves organizing the raw inertial data into a form usable by the other algorithms, applying frequency filters, and employing numerical differentiation. The second step examines the entire data set to determine the optimal non-varying joint center estimate via the variance minimization method, which will be explained in the coming sections. The third step uses the previously determined non-varying joint center estimate to stabilize an optimization process that calculates a joint center solution for each frame of data. The final step refines the results of the third step by applying a moving-average filter. Together, these four steps constitute the entirety of the SFO process. A flowchart representation of the SFO process is provided in Figure 2.

### 2.3. Data Preparation 

See Figure 1 for a complete layout of the data manipulation pre-optimization. The lowpass filter was a fourth-order, Butterworth filter applied bi-directionally to prevent phase change (raising the effective order from fourth to eighth). Referring to Equation (2), values for the translational acceleration and rotational velocity are obtained via direct measurement. Values for the angular acceleration cannot be obtained by measurement but can be well approximated by numerically differentiating ω. Many methods can be used to accomplish this, such as the three- or five-point differentiation methods. As the optimization stability could be affected by this, however, cubic splines are used to minimize differentiation error. The ω vectors are converted to piecewise cubic spline functions, which are then analytically differentiated and discretized at the appropriate time steps. 

### 2.4. Variance Minimization Method

Optimization requires the user to supply initial conditions to guide the optimization algorithm to a realistic solution. Poor initial conditions can greatly increase the required computation time, as well as lead to destabilization or the finding of a local minimum instead of a global one. The likelihood of one of these outcomes increases with cost function complexity (more roots provide more local minimums) and decreasing data quality (poor equation fitting destabilizes the search). Equation (2) makes for a reasonably complex cost function, but the true difficulty is the use of only a single data point that is most likely contaminated with some amount of STA. This complication makes the choice of initial conditions exceptionally important, as they will have a much greater effect on the calculated minimum than they would in traditional optimization, where there are many other data points to share the responsibility for convergence.

**Figure 3:**
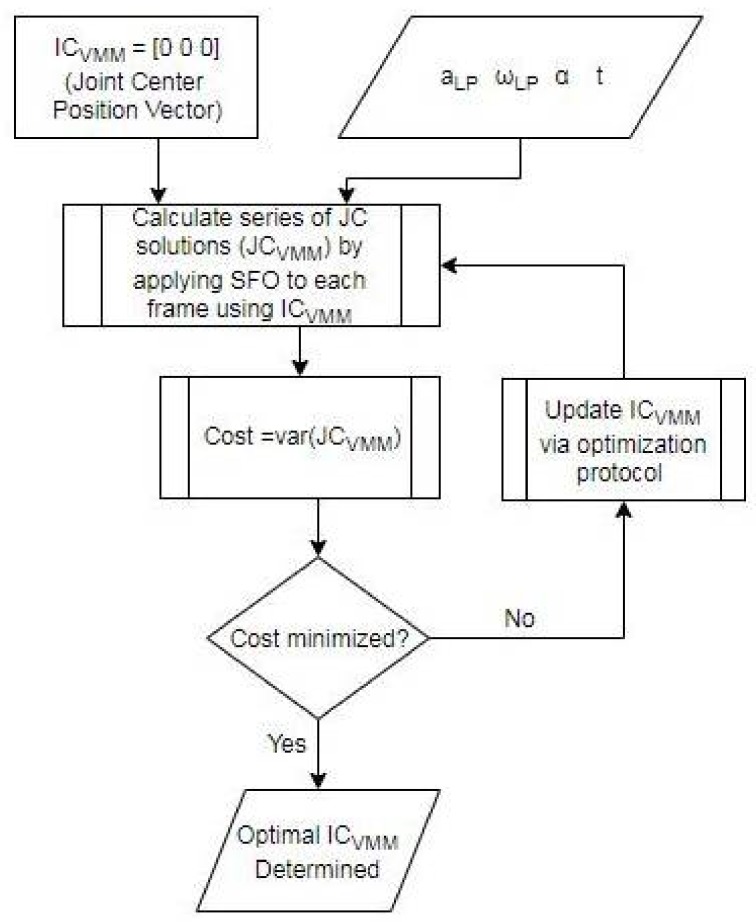
Flowchart detailing the variance minimization method process. The term “JC” denotes joint center, “var” denotes variance of, and “VMM” denotes variance minimization method.

A motivating factor for SFO’s development is the current inability to solve for joint centers accurately. Therefore, one would not expect users to accurately estimate reasonable initial conditions. Therefore, an unbiased method of objectively and reproducibly calculating them is needed. The method developed to fulfill this need is termed the “variance minimization method”, which operates by treating the variance of the array composed of each individual frame’s optimal joint center vector solution as its cost function. The overall initial conditions (used as the initial conditions for every frame’s optimization) are treated as the design variables. These overall initial conditions will henceforth be referred to as IC_VMM_ for clarity. Again, the Levenberg–Marquardt method is used with MATLAB’s default “fsolve” optimization settings. The only difference is that an analytical Jacobian is not provided. A flowchart detailing the variance minimization method process can be seen in Figure 3.

The variance minimization method operates under the assumption that the optimal IC_VMM_, meaning the position vector that most optimally fits the entire dataset, can be determined by minimizing the variance of the array composed of each individual frame’s optimization solution when using said IC_VMM_. Put simply, given the large number of time points considered, it is reasonable to assume that a non-negligible number of datasets was captured at times when the effects of STA were sufficiently negligible. These datasets would therefore be determined almost entirely by real motion. This is useful because the cost function defined by Equation (2) operates under the assumption that the input data consists of motion exhibiting a ratio of real motion to STA motion large enough for the STA to be relatively negligible. Spans of data points composed predominately of data with such a ratio can be referred to as high-quality data. By high-quality data, it is meant that when such a data point is subjected to the optimization process, the result will converge to the true solution regardless of the starting values of IC_VMM_. Let these high-quality data points be referred to as “constant points”. Meanwhile, the remaining, lower-quality data points will converge to solutions greatly influenced by the IC_VMM_. If the cost function is defined as the variance of the aforementioned array, minimization would be achieved by moving the IC_VMM_ closer to the constant point solution values. This movement would increase the quality of the IC_VMM_, allowing data points of quality slightly lower than the constant points to converge to their correct solutions. Repetition of this process eventually yields a vector value for IC_VMM_ that maximizes the number of data points stable enough for their optimization to converge to a reasonable solution. 

### 2.5. Comparator for SFO Validation

To contextualize the performance of the SFO process, it must be compared with an existing method. The method deemed most relevant for such a comparison is that propounded by Seel et al., henceforth referred to as Seel’s optimization method (SOM) [28,31]. As with SFO, SOM uses inertial sensors, subsumes the calibration process, and attempts to solve for the joint centers directly (rather than statistically). SOM was developed for use in human motion capture, so its driving cost function was applied so that it could deal with a joint center undergoing acceleration. This requires two sensors, one on either side of the joint center in question, and simultaneous solution of both joint center vectors. When SOM is simplified to be comparable with SFO as it has been developed thus far, its cost function reduces to that shown in Equation (2). In the optimization process, every data point is input to the cost function at the same time, resulting in a vector-valued cost-function solution. The single joint center solution that minimizes said vector is considered to be optimal. The optimization details for the SOM mirror those previously specified for SFO using the Levenberg–Marquardt algorithm within MATLAB’s “fsolve” function.

### 2.6. Numerical Examples

The SFO algorithm was tested by digitally replicating situations that would occur if an inertial sensor were attached to a rigid, articulating, planar pendulum. White Gaussian noise was added to the simulation’s raw inertial data (after any simulated STA was included in the data), and the noise’s magnitude was based on the noise specifications of the experimental sensors intended to be used in experimental testing, the XSENS Motion Tracker wireless IMU [34]. The noise was generated based on the values specified as the desired standard deviations, which were $0.0076\;m/s^{2}$ and $2.75\times 10^{-5}\;\mathrm{rad}/s$ for the translational acceleration and rotational velocity, respectively. 

**Figure 4:**
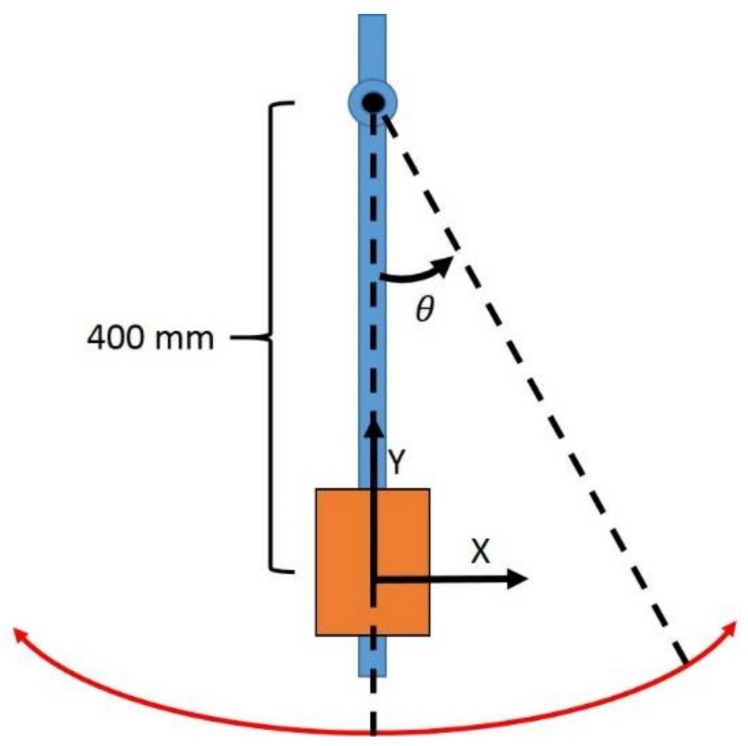
Diagram of numerically simulated pendulum setup. Note that the Z axis is perpendicular to the XY plane as defined by the right-hand rule. The orange block represents the inertial sensor.

The pendulum’s link length was set at 400 mm, a distance similar to typical human link lengths, and the initial conditions chosen to describe the pendulum’s state before release (IC_pend_) were θ= 90° and ω=0 rad/s. The pendulum movement was determined by solving the appropriate equations of motion for a pendulum released and acted on only by the force of gravity. Said equations were expressed as differential equations and solved via the built-in MATLAB function “ode45”, which is based on an explicit Runge–Kutta formula. IC_pend_ was provided as the solver’s starting point [33]. A diagram of the pendulum setup can be seen in Figure 4. Gravity was set to $9.8065\;m/s^{2}$ globally downwards. The simulation was set to run for a time of 25.12 s, and the sample rate was set to 100 Hz.

**Figure 5:**
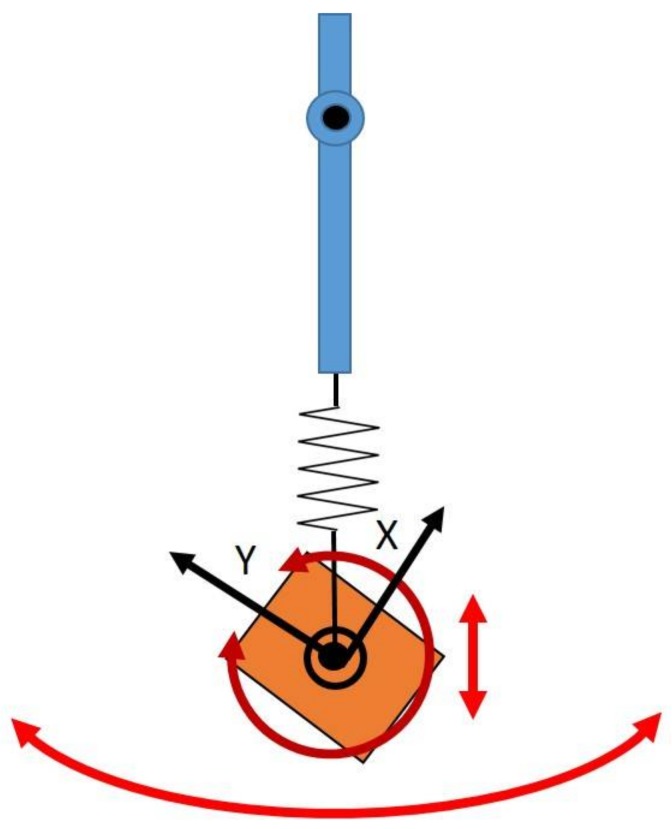
Diagram showcasing the modifications made to the simulated pendulum to achieve a better approximation of STA. The Z axis is perpendicular to the XY plane as defined by the right-hand rule.

The goal of the simulation is to assess the effect of STA on SFO. A major component of STA is relative translation, and this was reflected in the simulation by modeling the virtual IMU as attached to the pendulum via a spring (expanding and contracting in the direction of the link’s longitudinal axis) undergoing a set number of oscillations over the simulation’s time span (Figure 5). This scenario used the same conditions that were used to simulate the rigid link with only noise added, but the spring forced the length of the link to vary sinusoidally with a specifiable amplitude and frequency. The sinusoid’s period was set so that one oscillation was completed over the duration of the simulation. Adding this relative translation will force the SFO solution to change with each iteration. To account for this, the infrastructure of the SFO process is such that the initial conditions for SFO as applied to each frame, termed IC_SFO_, are defined by the solution obtained from applying SFO to the previous frame. Therefore, IC_VMM_ is used only once—as IC_SFO_ for the first data frame. The second data frame then uses the SFO solution from the first frame as the new IC_SFO_, repeating this process at each frame.

As shown in the literature, STA can contaminate human-motion measurements by introducing relative translation, rotation, scaling, shearing, and other deformations [23]. Furthermore, the literature suggests that the rigid components of STA (relative translation and relative rotation) are the components most relevant to the accuracy of kinematic calculations [17,21]. As such, the simulation was further modified to include a relative rotation component, and rotation of the IMU relative to its underlying structure was introduced (Figure 5). This was simulated by applying a series of rotation matrices to the simulated data, as well as updating the generated gyroscope measurements to reflect the added motion. For clarity, the chosen rotation matrices induced rotation about the Z axis only and caused two complete sinusoidal cycles of relative rotation with specifiable amplitude to occur over the simulation’s time span.

To supplement the four cases mentioned thus far, in which the STA was simulated by low-frequency sine waves, a fifth case was simulated that incorporated STA as a multimodal, higher-frequency sine wave. Specifically, the equations defining the relative translation and relative rotation are

(3) $$x_{spring}=0.5A_{t}\sin\left(0.25t\right)+0.1A_{t}\cos\left(2t\right)+0.025A_{t}\cos\left(4t\right)$$

(4) $$\theta_{rel}=0.5A_{\theta }\sin\left(0.125t\right)+0.25A_{\theta }\cos\left(0.5t\right)+0.05A_{\theta }\cos\left(2t\right)$$

where, $x_{spring}$ defines the simulated relative translation, and $\theta_{rel}$ defines the relative rotation. $A_{t}$ defines the amplitude of the relative translation and was set to 30 mm. $A_{rel}$ defines the amplitude of the relative rotation and was set to 2°. The frequencies used in the spring motion were 0.125, 2, and 4 Hz, while the frequencies used in the relative rotation were 0.125, 0.5, and 2 Hz. These frequencies were chosen because of the suggestion in the literature that the majority of the energy content for human motion is concentrated below 3.5 Hz [35,36], and because motion capture data is traditionally filtered via a Butterworth filter at 6 Hz [37]. The 0.125 Hz frequency was included to represent the motion of an IMU slowly changing its relative position during a motion trial, as, in the authors’ experience, commonly occurs when the IMUs are held in place by Lycra suits.

With the addition of the multimodal STA case, a total of five cases were simulated:

1. Rigid link with added noise (control simulation)
2. Rigid link with added noise and spring motion
3. Rigid link with added noise and relative rotation
4. Rigid link with added noise, spring motion, and relative rotation
5. Rigid link with added noise, multimodal spring motion, and multimodal relative rotation

In the following section, SFO and SOM are applied to each of the five aforementioned cases, and their results are compared both graphically and in terms of root mean square error (RMSE). Pearson correlation coefficient values for the SFO results are presented as well. The simulation was performed in planar settings, so only error in the two relevant axes (X and Y) was considered, as the third can correctly assume infinite values along the pendulum’s axis of rotation. For each method, the norm of the error was calculated at each point from these two axes, and the RMSE was calculated by taking the root mean square value of each vector of error norms. The correlation values were reported only if their associated *p*-values were significant (p<0.05).

## 3. Results

**Figure 6:**
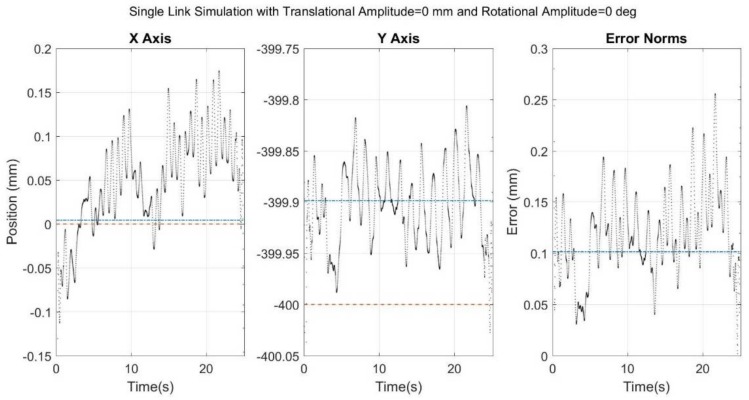
Results for simulation run with only noise added to a rigid link. Black dots refer to SFO, blue dash-dots to SOM, and red dashes to the theory.

**Figure 7:**
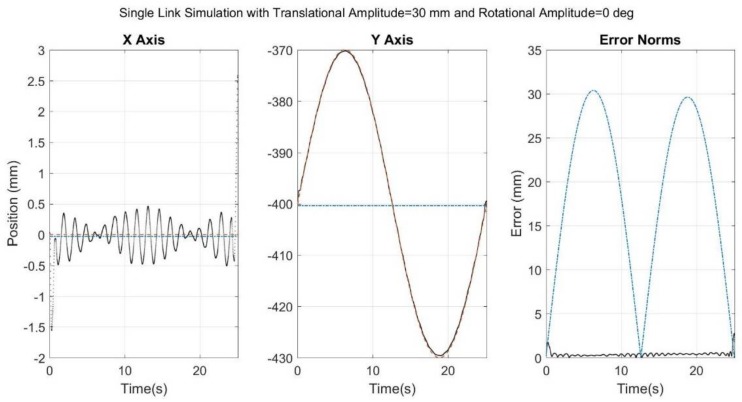
Results for simulation run with noise added to the rigid link as well as spring-induced relative translational motion of the sensor. Black dots refer to SFO, blue dash-dots to SOM, and red dashes to the theory. Note that the SFO and theory results exhibit significant overlap.

**Figure 8:**
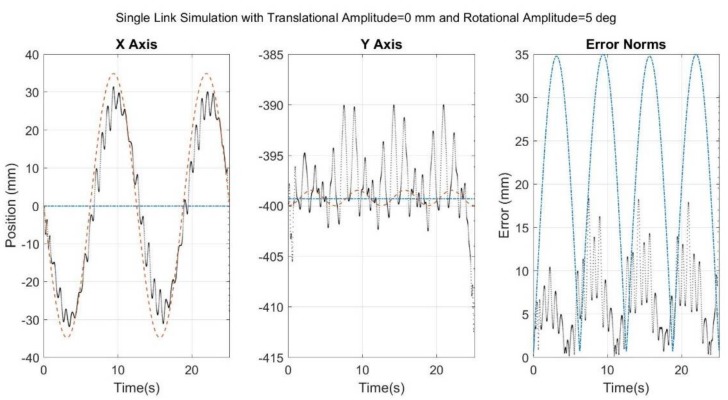
Results for simulation run with noise added to a rigid link as well as induced relative rotational motion of the sensor. Black dots refer to SFO, blue dash-dots to SOM, and red dashes to the theory.

**Figure 9:**
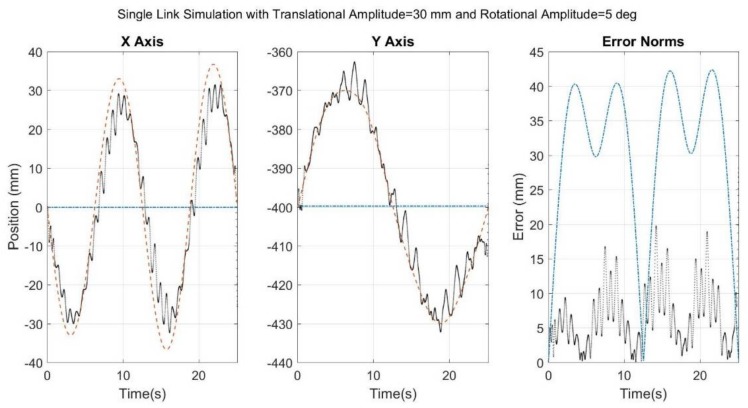
Results for simulation run with noise added to a rigid-link, spring-induced relative translational motion, and an induced relative rotational motion. Black dots refer to SFO, blue dash-dots to SOM, and red dashes to the theory.

**Figure 10:**
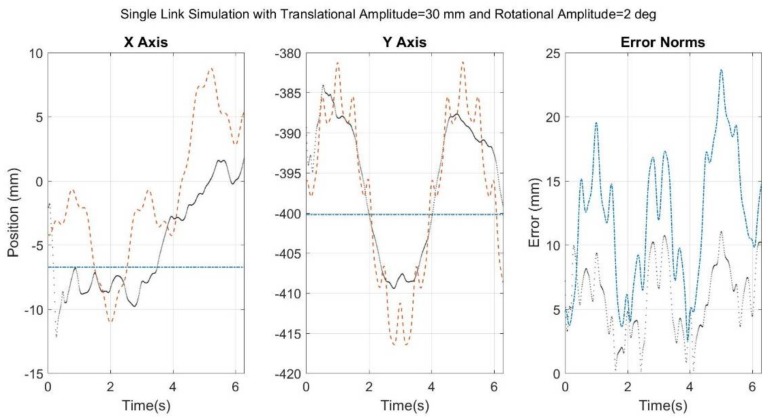
Results for simulation run with noise added to the rigid link, as well as the multimodal implementations of translational and rotational STA, as defined by Equations (3) and (4). Black dots refer to SFO, blue dash-dots to SOM, and red dashes to the theory.

Figure 6, Figure 7, Figure 8, Figure 9 and Figure 10 below showcase the results of the five simulation cases. In each figure, the left and middle images refer to the joint center vector for the X and Y axes, respectively. The image on the right is the overall error norm as a function of time. The black dotted plots denote SFO results, the blue dash-dot plots denote SOM results, and the red dashed plots denote theory results, meaning the true solution.

In the first control case, the results were as expected, with both SFO and SOM obtaining sub-mm accuracy (Figure 6). As the noise added was white and Gaussian, the optimization algorithms were well equipped to mitigate that potential source of error, and the ideal environment within the simulation maximized the optimization’s stability.

The results for the second case (Figure 7), specifically those of the Y axis plot, showcase the potential for SFO to outperform SOM. SFO’s adaptive solution caused a negligible amount of error in the X axis (less than ~0.5 mm) but outperformed SOM by up to 30 mm in the Y axis, which was the axis containing simulated STA. SOM was restricted to finding a single optimal vector response for the motion as a whole, and therefore its solution was unable to reflect the true motion’s sinusoidal behavior.

As with the results shown in Figure 7, the results for the third case (Figure 8) showed that SFO’s adaptability allowed it to track the sinusoidal nature of the motion. The major difference in this simulation case was the quality of the tracking, specifically the seemingly lower stability of optimization solutions. These results suggest that 5° of relative rotation destabilizes the optimization far more than 30 mm of relative translation.

The results for the fourth case (Figure 9) look much like a superposition of the results from Figure 7 and Figure 8. Given that the simulated STA for Figure 9 is a superposition of the STA simulated in Figure 7 and Figure 8, this suggests that a somewhat linear relationship may exist between the simulated STA and the resulting error, at least within the ranges of the simulated values. 

The results of the fifth case are seen below in Figure 10. Notice that the time scale has been shortened to slightly more than 6 s to allow for closer inspection of the effects caused by the higher-frequency simulated STA.

Figure 10 shows that SFO has greater difficulty in tracking these higher-frequency components than the lower-frequency ones, as was expected. However, SFO tracks the overall motion better than SOM does, both in terms of RMSE and in correlation to the theory. SOM did not lose accuracy because of the higher-frequency components; this was expected because the higher frequencies have little to no effect on the overall average relative motion.

The above results (Table 1) show that in all cases where STA was simulated, SFO produced RMSE values far lower than those produced by SOM. As for the control case where STA was not simulated, the error of both methods was negligible. Considering the correlation values, SFO exhibited high correlation values (*r* > 0.82) in all cases where STA produced significant changes in the joint center vector component. This indicates that SFO is capable of capturing the motion caused by the simulated STA.

## 4. Discussion

The method described in this paper, termed single-frame optimization (SFO), proposes a method of mitigating the effects of STA on the calculations of joint centers. SFO accomplishes this by representing and solving for the joint center vector, meaning the vector relating the joint center location to the location of an IMU within the IMU’s local coordinate system, in a time-varying manner. The time-varying joint center vector is determined by an optimization process that calculates an optimal rigid-body realization for each frame. These rigid-body realizations effectively linearize the joint center solution, capturing the motion due to STA. The effect of STA on the inertial measurements can then be quantified and mitigated, thereby improving calculation of joint kinematics.

### 4.1. Interpreting SFO Results

The simulation results for Cases 1–4 suggest that the SFO approach can accurately capture changes in the position vector, as is the method’s primary goal. These results also suggest that STA in the form of relative rotation negatively impacts SFO’s accuracy more than STA in the form of relative translation. The most likely explanation for this is that relative translation impacts only the translational acceleration, while relative rotation impacts the rotational velocity, rotational acceleration, and translational acceleration (i.e., changes its distribution along the axes but not its magnitude).

**Figure 11:**
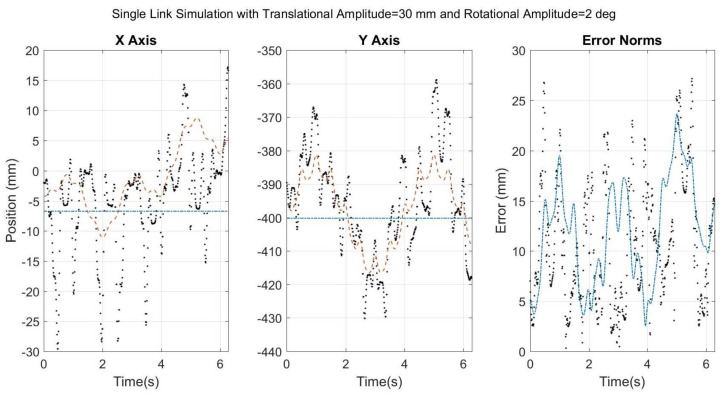
The same results as presented in Figure 10, but without frequency or moving average filtering applied to the SFO results. Black dots refer to SFO, blue dash-dots to SOM, and red dashes to the theory.

It is obvious from the time scale that the STA simulated in Cases 1–4 is not realistic. Therefore, the results of Case 5 (Figure 10) are likely more indicative of how SFO would respond to true STA. The results suggest that SFO can produce viable results for a more realistically simulated STA, but also that higher frequencies are more difficult to capture. Furthermore, the correlation values for Case 5 ($r_{X}=0.83$ and $r_{Y}=0.90$) suggest that, despite the higher-frequency content, the overall motion was well represented by the solution. However, the results seen in Figure 10 have been passed through a lowpass filter and a moving average filter as part of the SFO process. The unfiltered results offer a different perspective on SFO’s efficacy and are shown in Figure 11. The resulting RMSE for SOM and the unfiltered SFO were 13.85 mm and 14.93 mm, respectively, and the SFO correlation values were 0.48 and 0.72 for the X and Y axes, respectively.

The results without filtering explicitly show the potential for instability of individual SFO results, e.g., the errors in excess of 30 mm in the X axis. This instability is the price paid for attempting to linearize behavior that is highly nonlinear. In the Y axis, SFO does a far better job of tracking the STA, and this is likely partially the result of the Y axis motion being larger than that of the X axis and not being centered near zero.

Upon comparison with the filtered results (Figure 10), it is clear that a large portion of this instability can be mitigated by a simple filtering process. This suggests that the raw optimization data contains valuable information despite its scattered appearance. It also suggests that there is much room for improvement in how the raw data is processed. This could be as simple as applying the frequency and moving-average filters with adaptable parameters, or as complex as using an extended Kalman filter. There is much information currently unused by SFO that is potentially beneficial, such as the residual value of the objective function at each time point, the necessity of continuity of positional change, and the known cyclical nature of certain motions, such as human gait. 

### 4.2. Comparison with SOM

The major difference between SFO and SOM is that SFO produces a time-varying joint center vector, whereas SOM produces a single joint center vector. As a result, SFO can capture STA-induced changes in the joint center vector. It should be noted, however, that SOM could be applied multiple times within a given time frame, and that it requires only that the motion considered for optimization be rich enough to manifest relevant kinematic constraints [28]. Perhaps the SOM approach could be modified to detect data richness and update every time sufficient data has been gathered, or perhaps it could be applied via a moving-window approach. If these changes were made, SOM could be more adaptable to STA, and its associated RMSE would decrease. In the case of the moving-window application, the SOM solution would adapt to STA-induced changes, such as a sensor slowly sliding around during motion, but it would only capture the average of the STA effects in that time span.

To supplement the comparison of SFO with SOM, the relative computational costs should be discussed. Such comparison is difficult, as both methods were implemented in a way that maximizes accuracy rather than efficiency. This is particularly relevant in the case of the variance minimization method, due to its nested optimization structure. Therefore, the comparison will be broad. For the datasets simulated here (24 s), application of SFO (supplied with IC_VMM_) and SOM (to an entire dataset) required similar time to complete (approximately 1 s), though SOM was roughly one to two times faster. Determining the IC_VMM_, however, requires that the variance minimization method be applied to an entire dataset, and this required time periods between 5 min and 10 min. Therefore, as a whole, SFO required two to three orders of magnitude more time than SOM. The relevance of this, however, is potentially diminished when one considers that the time period required was less than 10 min, and that the variance minimization method has a high potential for efficiency improvement, were that deemed a priority.

### 4.3. Assumptions and Limitations

The literature shows that lowpass filters with cut frequencies of near 6 Hz are commonly used to filter human motion data [37,38]. Therefore, the STA component of the signal should be captured by the linearization process as long as the sample rate is two or more times the chosen cut frequency. A single XSENS Motion Tracker wireless IMU can produce sample rates of up to 120 Hz, so reaching the necessary sample rate should not be limited by the inertial hardware [34]. Despite this, there are some significant limitations to SFO that must be addressed.

The first and most important assumption that SFO makes is that STA motion can be considered negligible when compared to real motion. More specifically, it is assumed that the acceleration components induced by STA motion are negligible relative to the acceleration components induced by real motion. This is an assumption that will eventually need to be substantiated experimentally, but currently the authors believe it is a reasonable assumption for normal motion such as gait. However, there will certainly be instances when this assumption is not reasonable, such as during dynamic movements or when the real motion suddenly ceases (STA will almost certainly be active for a short while after such an occurrence). In cases such as these, where the aforementioned assumption is violated, it is clear that SFO will destabilize, meaning that the optimization process will converge to a non-viable minimum. As SFO only considers data from a single frame, it is unstable, and if the assumptions of its cost function are violated, it will almost certainly converge to a value that can clearly be identified as grossly inaccurate (a non-viable minimum). If this is not clear from the actual solution, it should be clear from the large residual value of the objective function at the calculated minimum. 

Because these results could be clearly identified as incorrect, they could be excluded from the final solution (as a gap). It could also be reasonably assumed that these unstable results indicate minimal real motion, and instead of a gap, the solution value just before the gap could be repeated through the gap. It is also worth noting that SFO is not the only method that suffers from this problem; all methods known to the authors, both optical and inertial, would suffer in some manner from such an occurrence. How well the occurrence is handled would depend on the method in use and the supplementary algorithms the method employs to deal with such situations. Developing a method for SFO that can assess the suitability of a given data set has been considered, as has a method for extracting information from data deemed unsuitable. For example, metrics such as the recent frequency content of the inertial measurements, the cost function value post-convergence, and the periodicity of motion in things like gait could all be leveraged to assess data suitability and/or improve the corresponding SFO solutions. 

The second limitation of the proposed method is its assumption/requirement that the acceleration of the joint center be negligible. The authors do not believe it is likely that there is any instance in human motion capture where this assumption would be adequately satisfied. Therefore, SFO, as presented, is likely not applicable to human motion capture. However, the authors intend to address this limitation in future work, and preliminary results suggest it can be overcome.

Pertaining to the third limitation, estimations of STA exist in the literature, and the simulated multimodal STA was based on these estimations [35,36]. However, these estimations were obtained for cases of marker-based motion capture, and it is not clear how valid they are in the case of inertial motion capture. The standard 14 mm optical marker (without base) weighs approximately 1.5 g, whereas an XSENS Motion Tracker wireless IMU weighs 27 g [34]. As for the attachment area, the same marker’s associated base was approximately 225 mm^2^, and the Motion Tracker’s base was approximately 1750 mm^2^ (nearly eight times larger). Even accounting for variability in marker and IMU choice, these differences must be considered. Furthermore, the estimations of STA in the current literature are primarily expressed in terms of position or orientation [10], and IMUs measure the second and first derivatives of these metrics respectively. Therefore, while the existing literature estimations of STA are an invaluable starting point for assessing the viability of SFO, they would be greatly supplemented by an inertial-sensor-specific characterization of STA or experimental data. 

There is a final limitation that does not apply to the SFO method, but rather to the environment in which it was simulated. As presented, there is no theoretical reason that SFO could not be applied to a 3D simulation rather than a planar one. The mathematical description of Equation (2) is such that it can be applied to joints modeled as having one, two, and/or three degrees of freedom. The reason for presenting a planar case is that it produces results that are far easier to understand, interpret, and critique than those of a 3D case. In a 3D case, the results must be expressed from all three relevant orientations instead of just one, complicating their interpretation. Furthermore, the determination of pendulum movement and relative rotation would be further complicated. This article is intended to act as proof of concept for the SFO method. As such, it was applied in a simple and easy-to-understand (planar) manner to ensure that it was critiqued on its most basic conceptual level. An unfortunate tradeoff, however, is that SFO has not been verified for 3D motion, a limitation that will be addressed in future work.

## 5. Conclusions

A new methodology for inertial motion capture, termed single-frame optimization, is presented in this work. The method attempts to mitigate soft-tissue artifact by calculating a time-varying joint center vector via an optimization process. To counter the instability posed by optimizing over a single time point, variance minimization is used (via a nested optimization approach) to calculate optimal initial conditions. The time-varying solution determined by this method allows the motion of soft-tissue artifact to be captured directly. When compared against a state-of-the-art inertial method via numerical simulation, the calculated error due to soft-tissue artifact was reduced in all cases (minimum reduction was 46%). Furthermore, the calculated time-varying solution was highly correlated (r>0.82) with the true solution in most cases. The proposed methodology, as currently presented, is intended as a proof of concept. The method needs to be tested under more realistic conditions in the future, including accelerating joint centers and spherical joints, before considering it for application in human motion.

## Figures and Tables

**Table 1:** Tabulation of the simulation results for SFO and SOM. $r_{X}$ denotes the Pearson correlation between the SFO X axis results and the theory X axis results. $r_{Y}$ denotes the same but for the Y axis. Only results with acceptable *p*-values (p>0.05) were displayed. The * symbol refers the reader to reference multimodal amplitude as defined by Equation (3), and the ** refers the reader to reference multimodal rotation as defined by Equation (4).

Simulated Case			SFO			SOM
	Amplitude (mm)	Rotation (°)	RMSE (mm)	$r_{X}$	$r_{Y}$	RMSE (mm)
Case 1	0	0	0.15	-	0.00	0.10
Case 2	30	0	0.474	-	1.00	21.22
Case 3	0	5	7.80	0.97	0.33	24.70
Case 4	30	5	7.83	0.97	0.99	32.53
Case 5	Multimodal *	Multimodal **	7.53	0.825	0.90	13.85
